# P-922. Feasibility and Diagnostic Performance of Sepsis Scores in Supporting Early Intervention and Strengthening Antimicrobial Stewardship in Low-Resource Hospitals in LMICs

**DOI:** 10.1093/ofid/ofaf695.1128

**Published:** 2026-01-11

**Authors:** Sushantika Chaudhary

**Affiliations:** Maharishi Markandeshwor University of Health sciences, ambala, Haryana, India

## Abstract

**Background:**

Diagnosing sepsis remains difficult due to its sudden presentation and the need for immediate clinical decisions. The SSC 2021 guidelines recommend SOFA as the gold standard, but its reliance on laboratory investigations limits use in low-resource hospitals. Other scores not requiring labs raise questions about diagnostic accuracy compared to SOFA. This study compares qSOFA, NEWS2, MEWS, and SOFA to identify a feasible and accurate screening method for early sepsis detection and analyse their association with culture positive in resource-limited settings.

**Figure:**
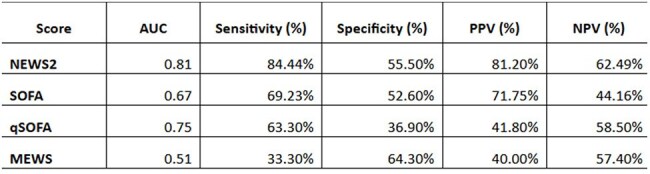
Diagnostic accuracy and comparison of Sepsis scores

**Figure:**
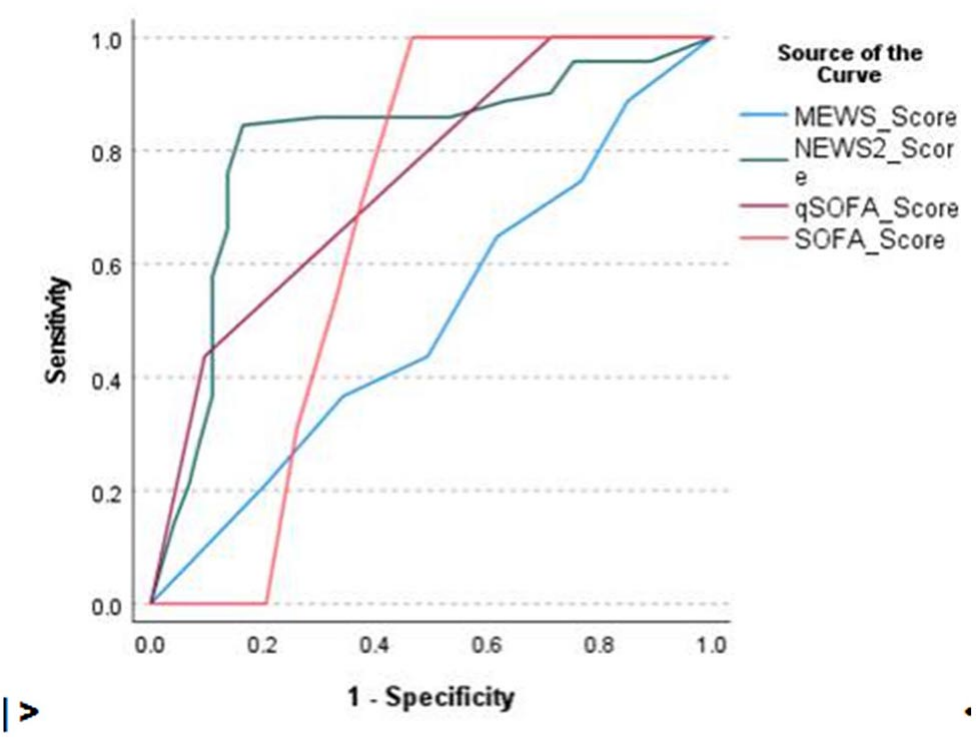
ROC curve

**Methods:**

A six-month prospective observational study was conducted among adult patients ( >18 years) with suspected infection presenting to the Emergency Department and ICU of a tertiary care hospital. qSOFA, SOFA, NEWS2, and MEWS scores were calculated at admission. ROC curve analysis and AUC comparisons were performed to evaluate diagnostic accuracy and chi square test for association of blood culture positives.

**Results:**

Of 411 patients with suspected or confirmed infection, 144 with sepsis were included. Among the scores, NEWS2 showed the highest diagnostic accuracy with AUC 0.81, sensitivity 84.4% and PPV 81.2%. (fig 1) SOFA had moderate performance with AUC 0.67, while qSOFA showed better sensitivity with AUC 0.75 but lower specificity. MEWS performed the poorest with AUC 0.51. (Table 1). NEWS2 greater than 7 was significantly associated with blood culture positivity with a p-value of 0.032, supporting its use in guiding antimicrobial initiation.

**Conclusion:**

This study shows that NEWS2, based solely on vital signs, can reliably identify sepsis with diagnostic accuracy comparable to more complex scores, offering a practical solution for early detection in low-resource settings. A NEWS2 score > 7 demonstrated significant association with blood culture positivity, supporting its use to guide early antibiotic initiation where laboratory support is limited. These findings represent an important step toward improving sepsis care and antibiotic stewardship in frontline environments. Further validation is needed, and NEWS2 should be considered for adoption as a standard tool for sepsis diagnosis in low-resource healthcare settings

**Disclosures:**

All Authors: No reported disclosures

